# Supplementary material for: Chemical synthesis of peptidoglycan mimetic–disaccharide-tetrapeptide conjugate and its hydrolysis by bacteriophage T5, RB43 and RB49 L-alanyl-D-glutamate peptidases
Source: PeerJ. 2021 May 19;9:e11480. doi: 10.7717/peerj.11480 (PMC8140593; doi:10.7717/peerj.11480)
Supplement: Supplemental Information 1 [file peerj-09-11480-s001.pdf]

SUPPORTING INFORMATION

Chemical Synthesis of Peptidoglycan Mimetic – Disaccharide-Tetrapeptide Conjugate and its Hydrolysis with Bacteriophage T5, RB43 and RB49 L-Alanyl-D-Glutamate Peptidases

Viatcheslav N. Azev[a], Alexey N. Chulin[a], Maxim M. Molchanov[b], Dmitry A. Prokhorov[b], Galina V. Mikoulinskaia[a], Vladimir N. Uversky[c], Viktor P. Kutyshenko[b,*]

[a] Branch of Shemyakin and Ovchinnikov Bioorganic Chemistry Institute, Russian Academy of Sciences, Science avenue, 6, Pushchino, Moscow Region, Russia, 142290.
[b] Institute for Theoretical and Experimental Biophysics, Russian Academy of Sciences, Institutskaya str., 3, Pushino, Moscow region, Russia, 142290.
[c] Department of Molecular Medicine and USF Health Byrd Alzheimer's Research Institute Morsani College of Medicine, University of South Florida, 12901 Bruce B. Downs Blvd. MDC07 Tampa, Florida 33612, USA.

[*] Correspondence to: Viktor P. Kutyshenko, Institute for Theoretical and Experimental Biophysics, Russian Academy of Sciences, Institutskaya str., 3, Pushino, Moscow region, Russia, 142290, *E-mail: kutyshenko@rambler.ru.*

1 NMR spectra and HPLC chromatograms

1 N_α-tert-butoxycarbonyl-L-alanyl-α-tert-butyl-D-glutamyl-L-alanyl-D-alanine tert butyl ester (**4**), ^{1}H NMR spectrum. 2
2 N_α-tert-butoxycarbonyl-L-alanyl-α-tert-butyl-D-glutamyl-L-alanyl-D-alanine tert butyl ester (**4**), {^{1}H}^{13}C NMR spectrum. 3
3 N_α-tert-butoxycarbonyl-L-alanyl-α-tert-butyl-D-glutamyl-L-alanyl-D-alanine tert butyl ester (**4**), 2D COSY NMR spectrum 4
4 N_α-tert-butoxycarbonyl-L-alanyl-α-tert-butyl-D-glutamyl-L-alanyl-D-alanine tert butyl ester (**4**), 2D HMBC NMR spectrum 5
5 HPLC analysis of L-Alanyl-γ-D-glutamyl-L-alanyl-D-alanine trifluoroacetate (**5**) 6
6 HPLC analysis of N-Acetylglucosaminyl-β-(1-4)-N-acetylmuramoyl-L-Alanyl-γ-D-glutamyl-L-alanyl-D-alanine (**1**). 7
7 N-Acetylglucosaminyl-β-(1-4)-N-acetylmuramoyl-L-Alanyl-γ-D-glutamyl-L-alanyl-D-alanine (**1**). ^{1}H NMR spectrum. 8
8 N-Acetylglucosaminyl-β-(1-4)-N-acetylmuramoyl-L-Alanyl-γ-D-glutamyl-L-alanyl-D-alanine (**1**). ^{13}C NMR spectrum. 9
9 N-Acetylglucosaminyl-β-(1-4)-N-acetylmuramoyl-L-Alanyl-γ-D-glutamyl-L-alanyl-D-alanine (**1**). HRMS spectrum. 10

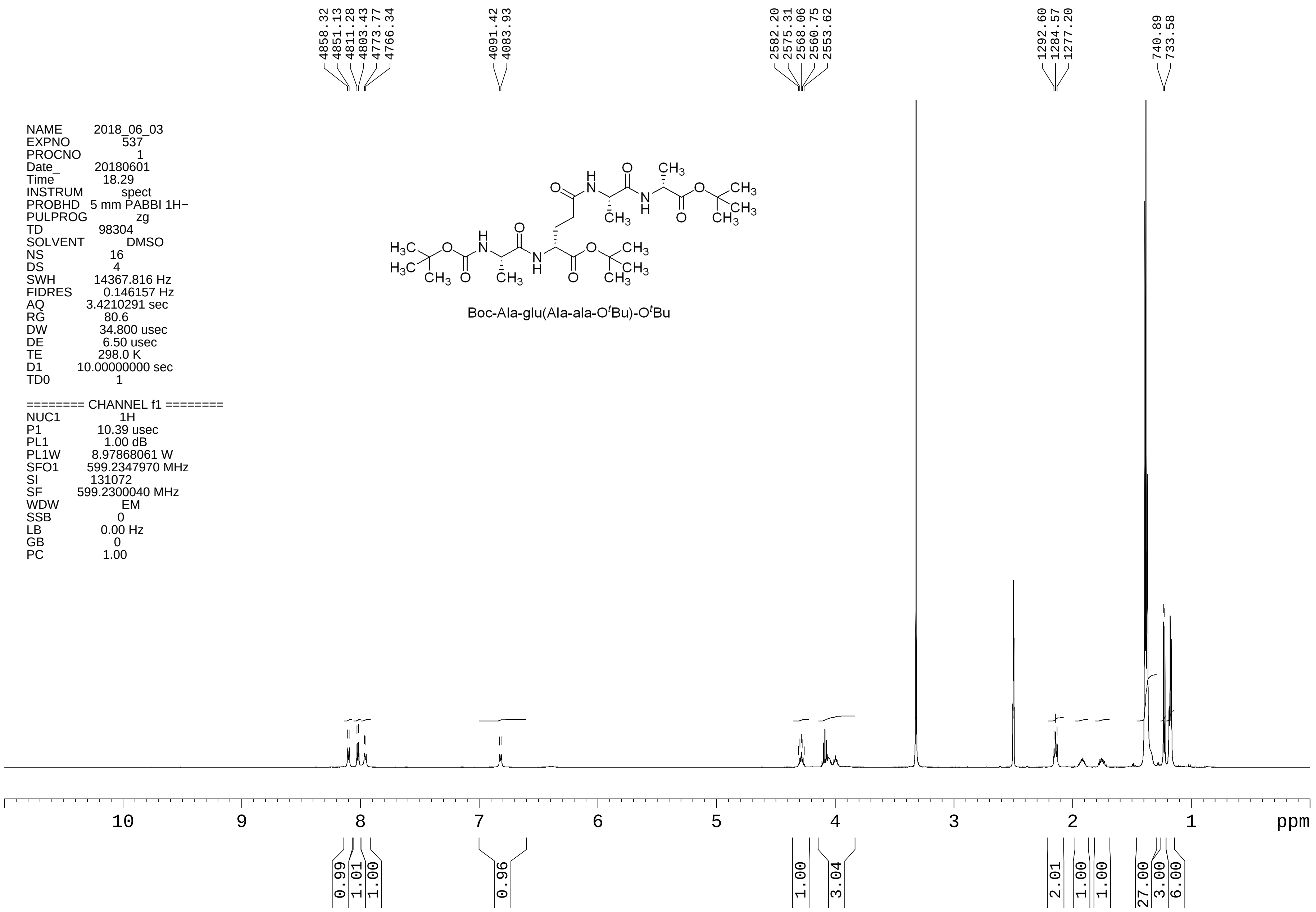

Figure 1: N$_\alpha$-tert-butoxycarbonyl-L-alanyl-α-tert-butyl-D-glutamyl-L-alanyl-D-alanine tert butyl ester (**4**), ^{1}H NMR spectrum.

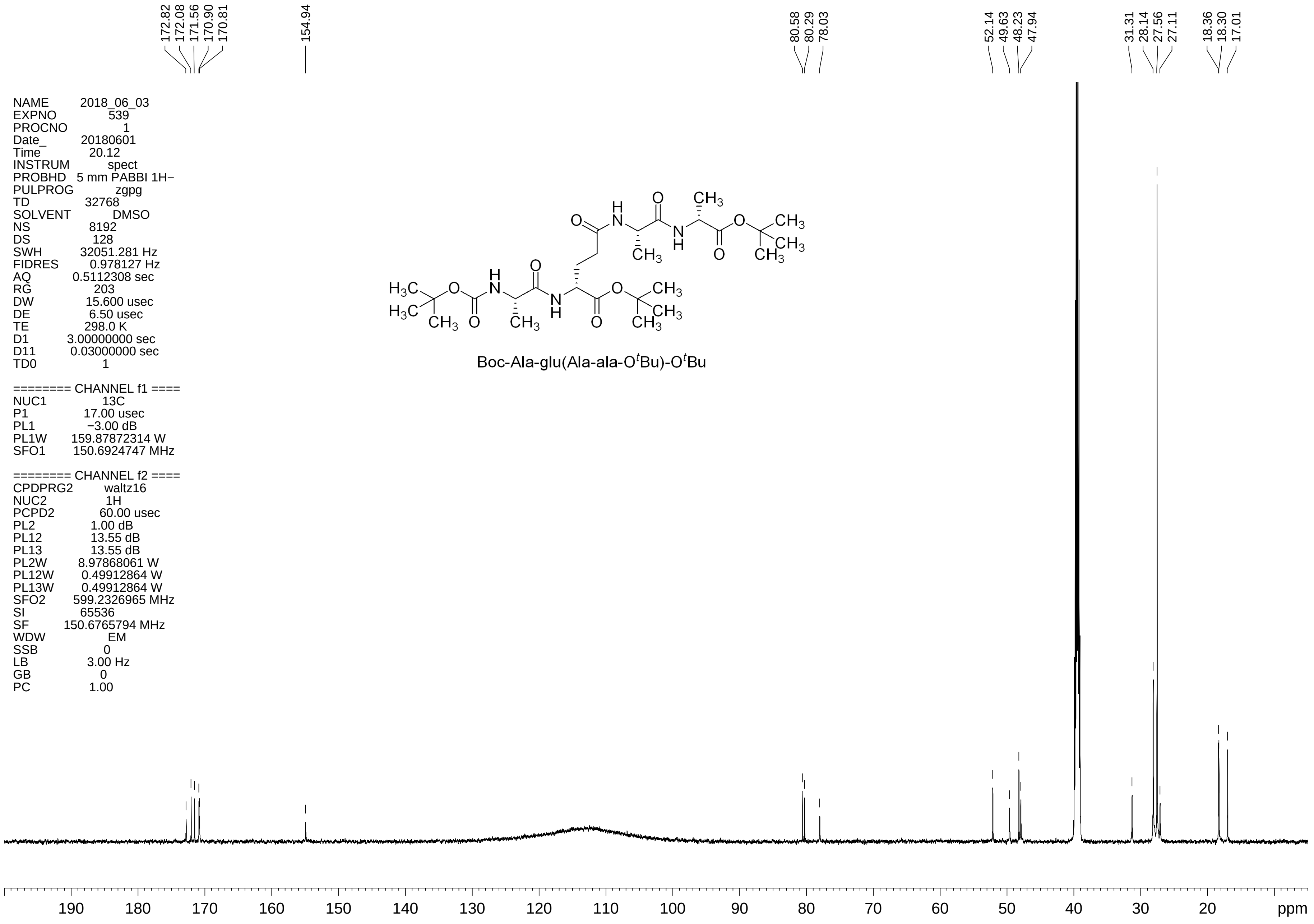

Figure 2: N$_\alpha$-tert-butoxycarbonyl-L-alanyl-α-tert-butyl-D-glutamyl-L-alanyl-D-alanine tert butyl ester (4), $\{^1H\}^{13}C$ NMR spectrum.

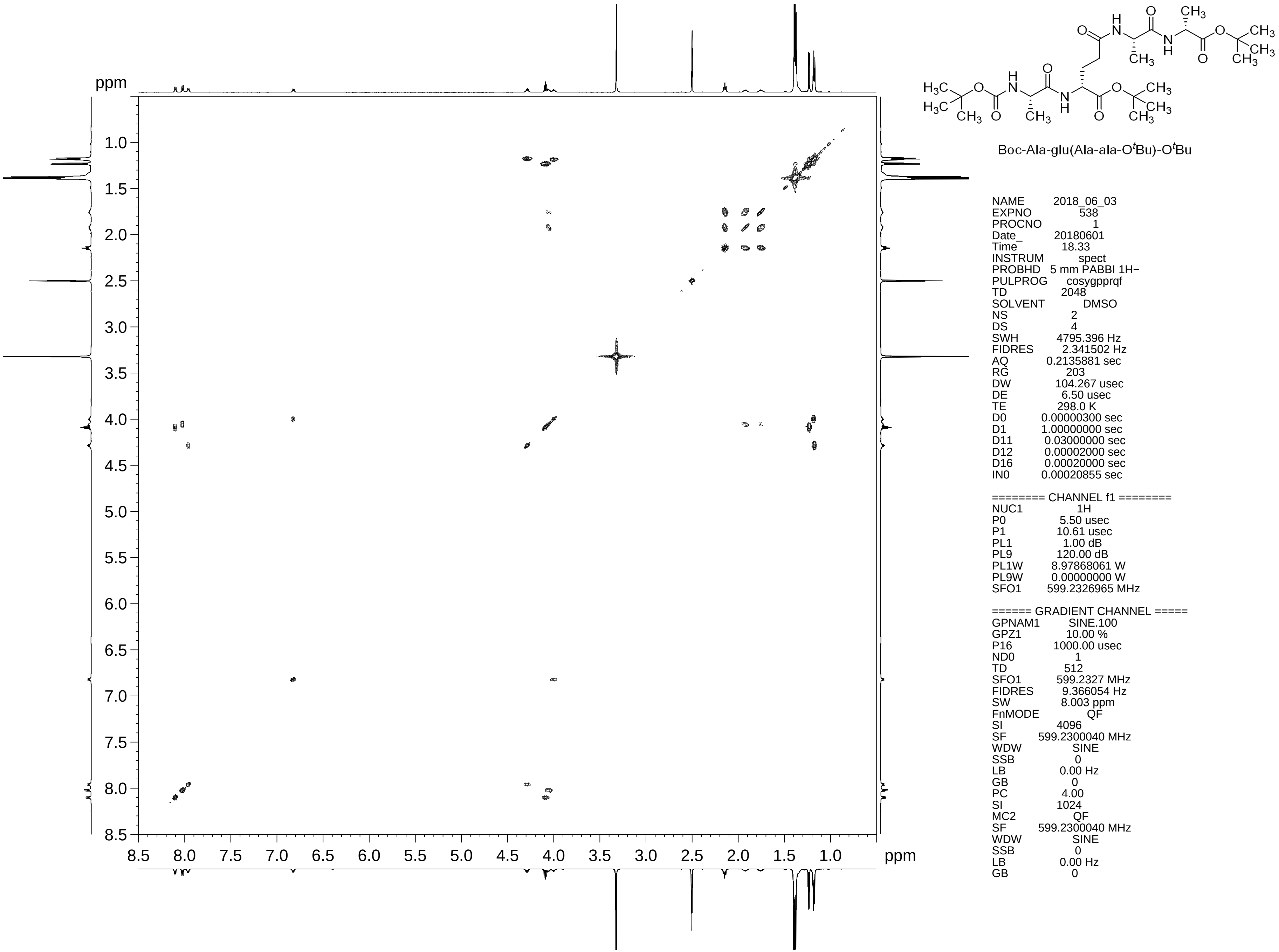

Figure 3: N_α-tert-butoxycarbonyl-L-alanyl-α-tert-butyl-D-glutamyl-L-alanyl-D-alanine tert butyl ester (**4**), 2D COSY NMR spectrum

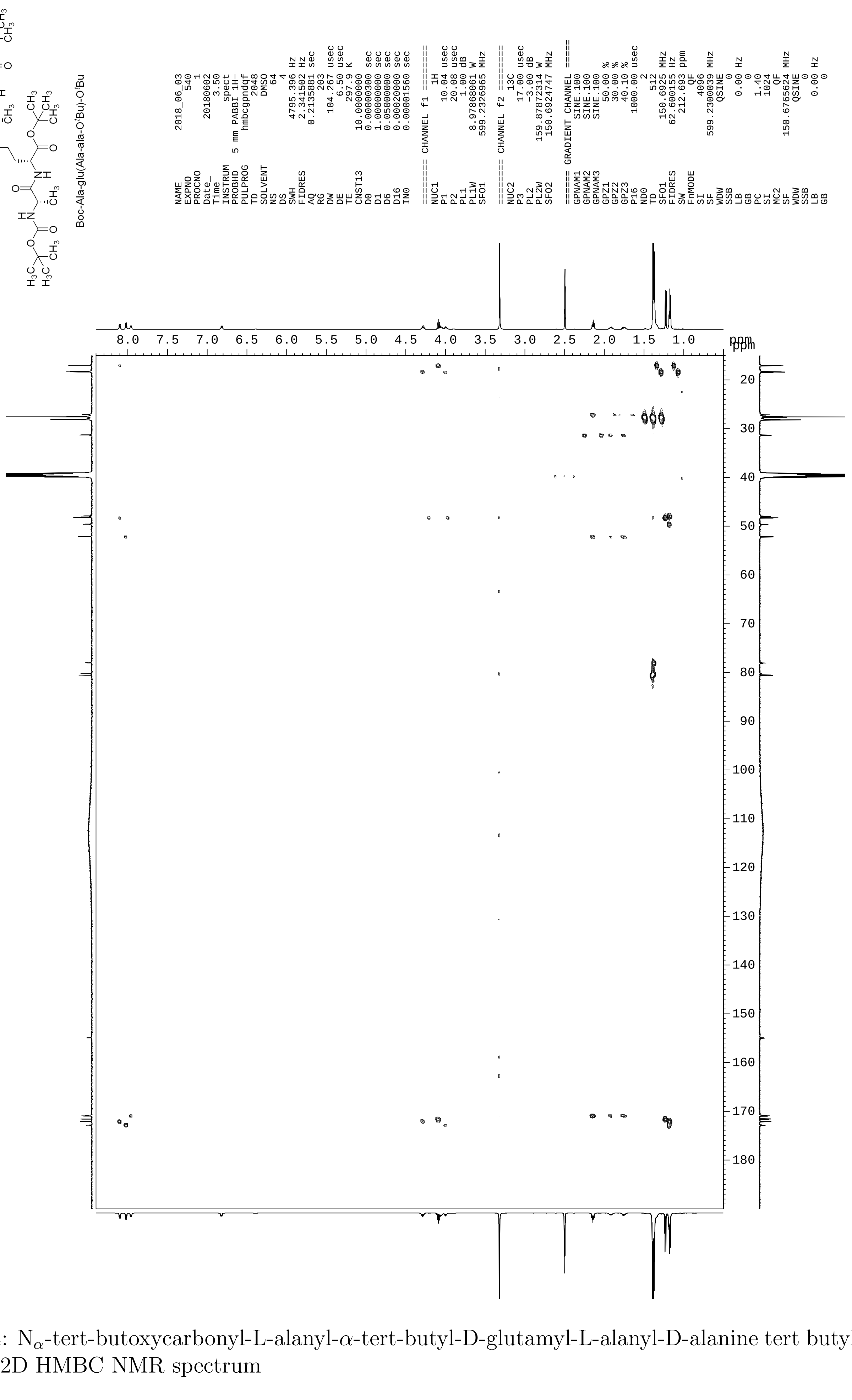

Figure 4: N$_\alpha$-tert-butoxycarbonyl-L-alanyl-α-tert-butyl-D-glutamyl-L-alanyl-D-alanine tert butyl ester (**4**), 2D HMBC NMR spectrum

Project Name: testing_peptides
Reported by User: System

SAMPLE INFORMATION

Sample Name:	Ag(Ag)	Acquired By:	System
Sample Type:	Standard	Date Acquired:	15.06.2018 14:29:03
Vial:	1	Acq. Method:	gradient1_0_55_25min
Injection #:	4	Date Processed:	24.03.2020 14:09:31
Injection Volume:	50,00 ul	Channel Name:	2487Channel 1
Run Time:	25,00 Minutes	Channel Desc.:	226
Column Type:		Sample Set Name:	

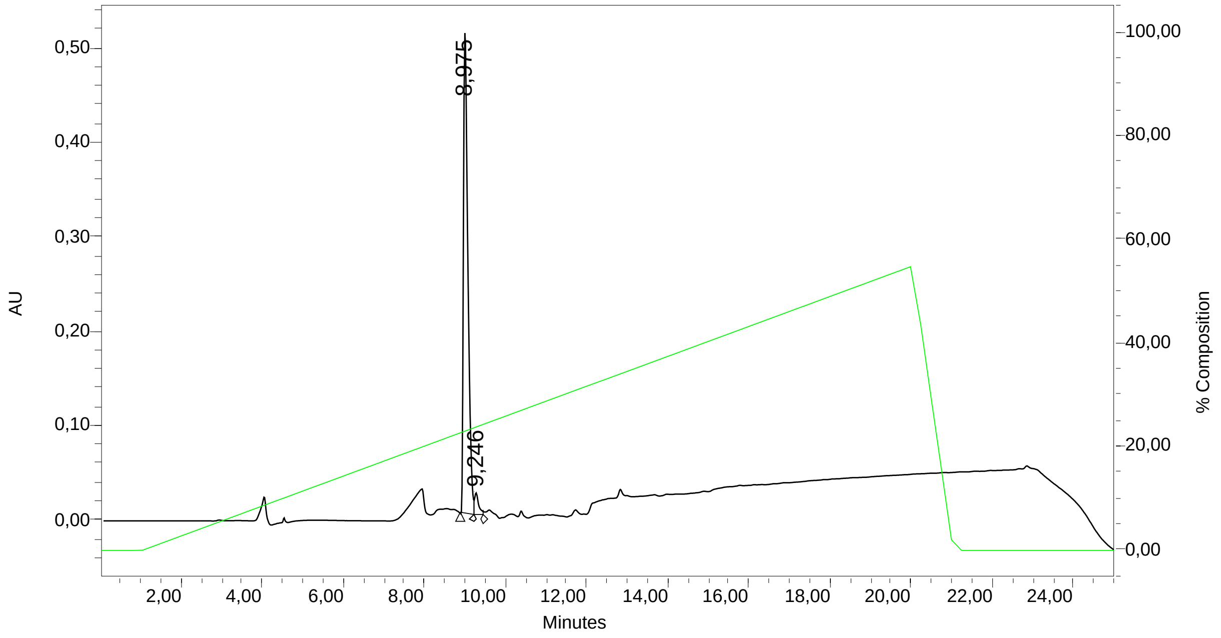

	RT (min)	Area (µV*sec)	% Area	Height (µV)	% Height
1	8,975	3904417	95,40	515390	95,57
2	9,246	188377	4,60	23872	4,43

Report Method: Gradient Overlay Report ASC Printed 14:09:41 24.03.2020 Page: 1 of 1

Figure 5: HPLC analysis of L-Alanyl-γ-D-glutamyl-L-alanyl-D-alanine trifluoroacetate (**5**)

Project Name: testing_peptides
Reported by User: System

SAMPLE INFORMATION

Sample Name:	ppt2 C18, 6% AcCN	Acquired By:	System
Sample Type:	Standard	Date Acquired:	22.01.2019 13:37:41
Vial:	1	Acq. Method:	grad_3_2m_50_226_280
Injection #:	7	Date Processed:	24.03.2020 13:33:52
Injection Volume:	50,00 ul	Channel Name:	2487Channel 1
Run Time:	25,00 Minutes	Channel Desc.:	226 nm
Column Type:		Sample Set Name:	

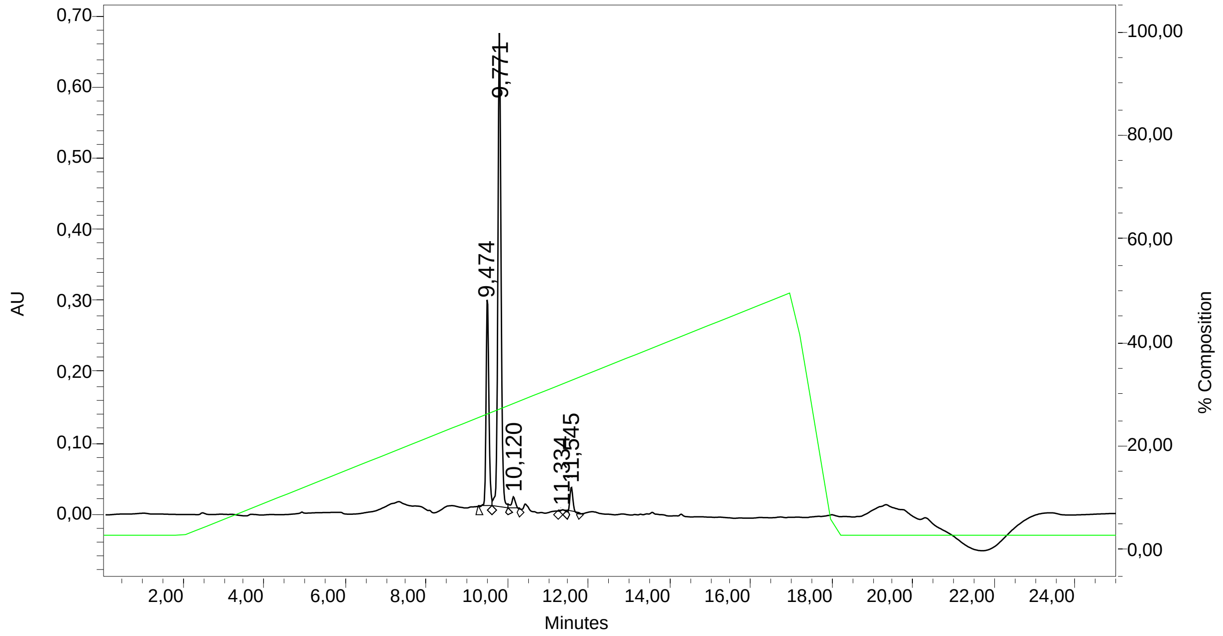

	RT (min)	Area (µV*sec)	% Area	Height (µV)	% Height
1	9,474	1449587	26,46	291774	28,94
2	9,771	3722905	67,96	662888	65,74
3	10,120	103782	1,89	16464	1,63
4	11,334	16751	0,31	2644	0,26
5	11,545	184791	3,37	34560	3,43

Report Method: Gradient Overlay Report ASC Printed 13:34:08 24.03.2020 Page: 1 of 1

Figure 6: HPLC analysis of N-Acetylglucosaminyl-β-(1-4)-N-acetylmuramoyl-L-Alanyl-γ-D-glutamyl-L-alanyl-D-alanine (**1**). Note: compound **1** posesses an anomeric center and hence it exists as a mixture of diastereomers: peaks at 9.47 and 9.77 min.

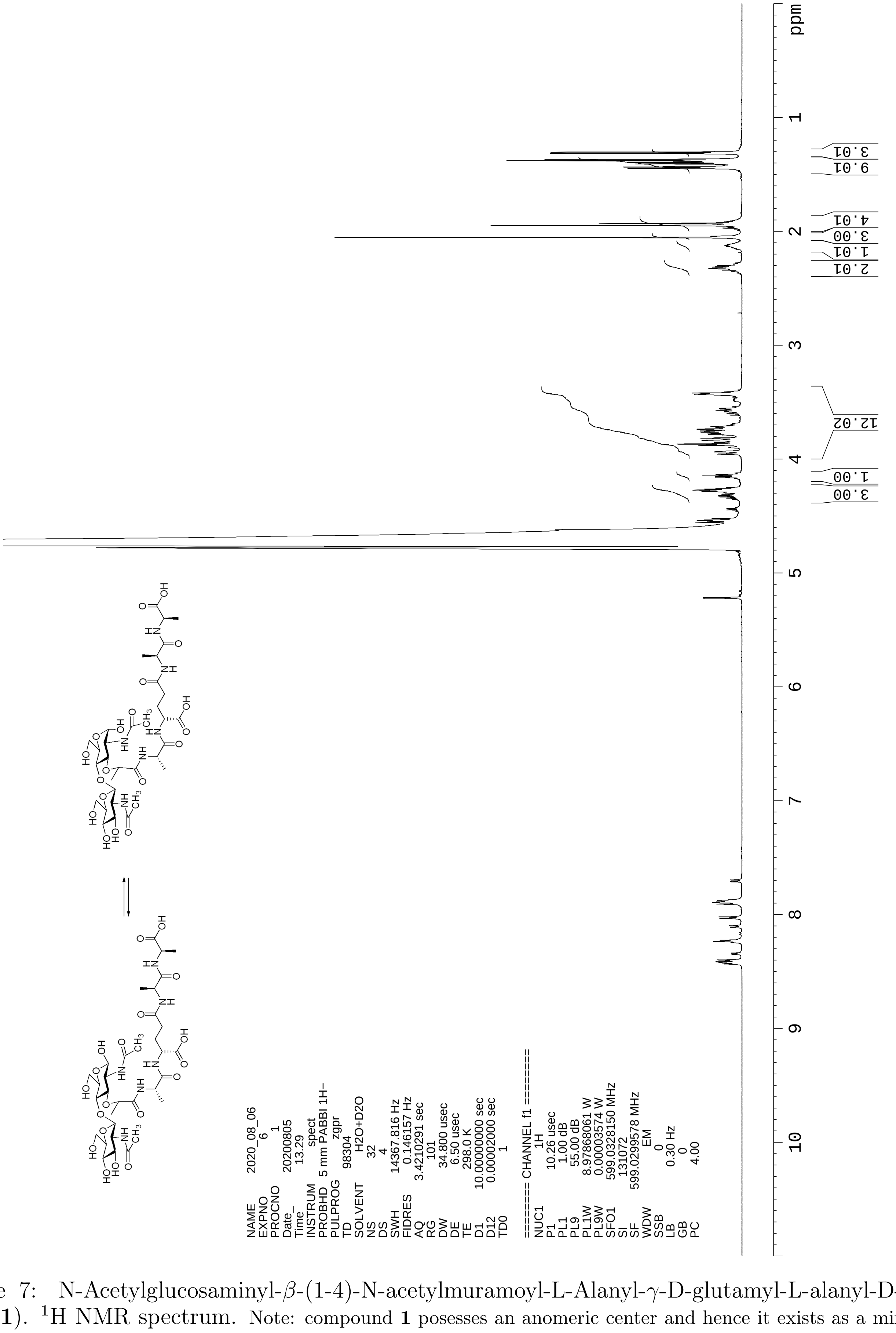

Figure 7: N-Acetylglucosaminyl-β-(1-4)-N-acetylmuramoyl-L-Alanyl-γ-D-glutamyl-L-alanyl-D-alanine (**1**). ^{1}H NMR spectrum. Note: compound **1** posesses an anomeric center and hence it exists as a mixture of diastereomers. Therefore, only partial integration of proton signals was made. At the same time, the result of integration does support the fact that compound **1** exists as an adduct of one molecule of disaccharide and one molecule of tetrapeptide. For complete signal assignment, that also supports the nature of the linkage between the disachraride unit and the tetrapeptide, see manuscript text. Also, no impurities signals with normalized areas larger than 5 % except residual solvent are observed, hence compound **1** does attain purity requirments.

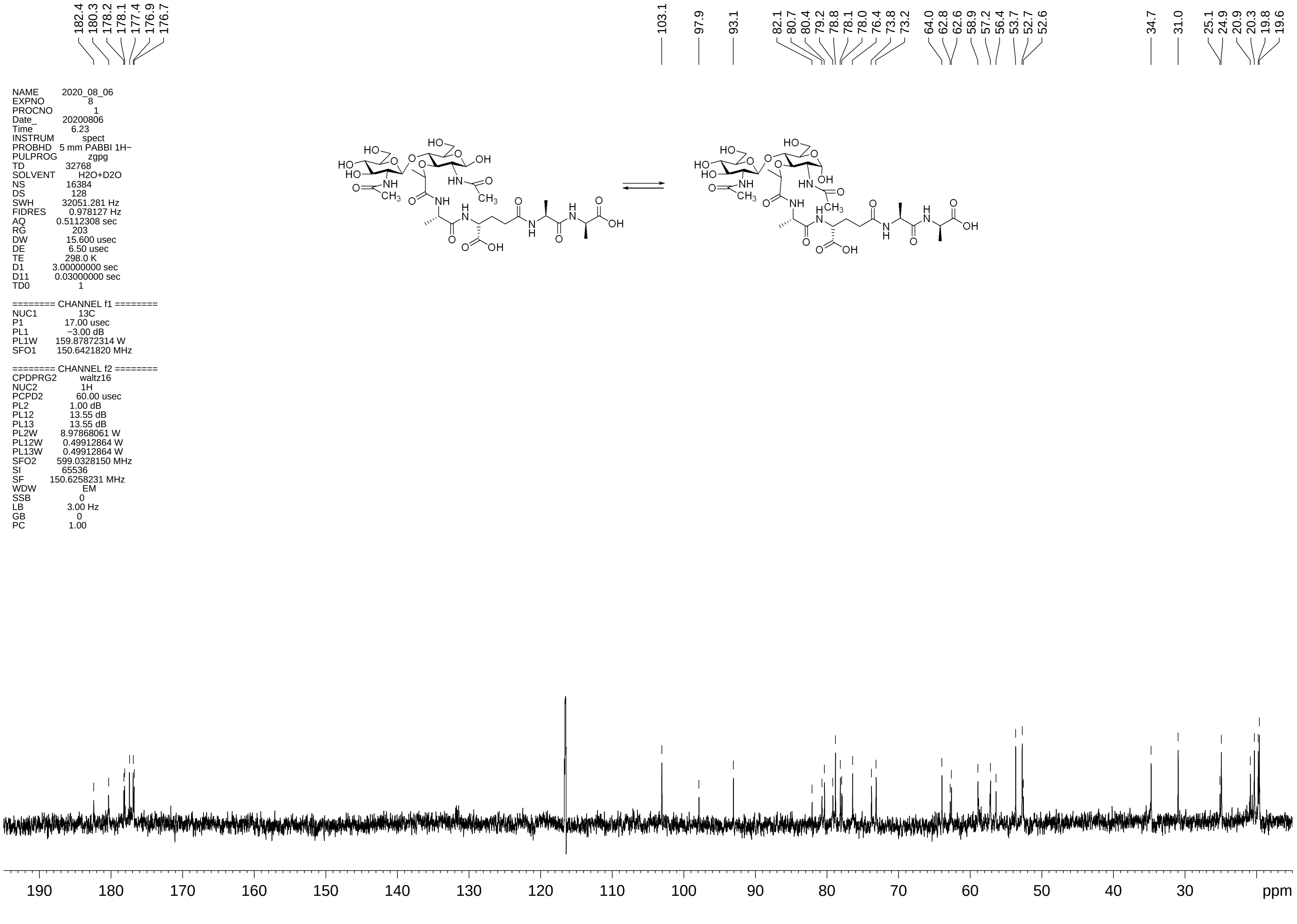

Figure 8: N-Acetylglucosaminyl-β-(1-4)-N-acetylmuramoyl-L-Alanyl-γ-D-glutamyl-L-alanyl-D-alanine (**1**). ^{13}C NMR spectrum. For complete signal assignment see manuscript text.

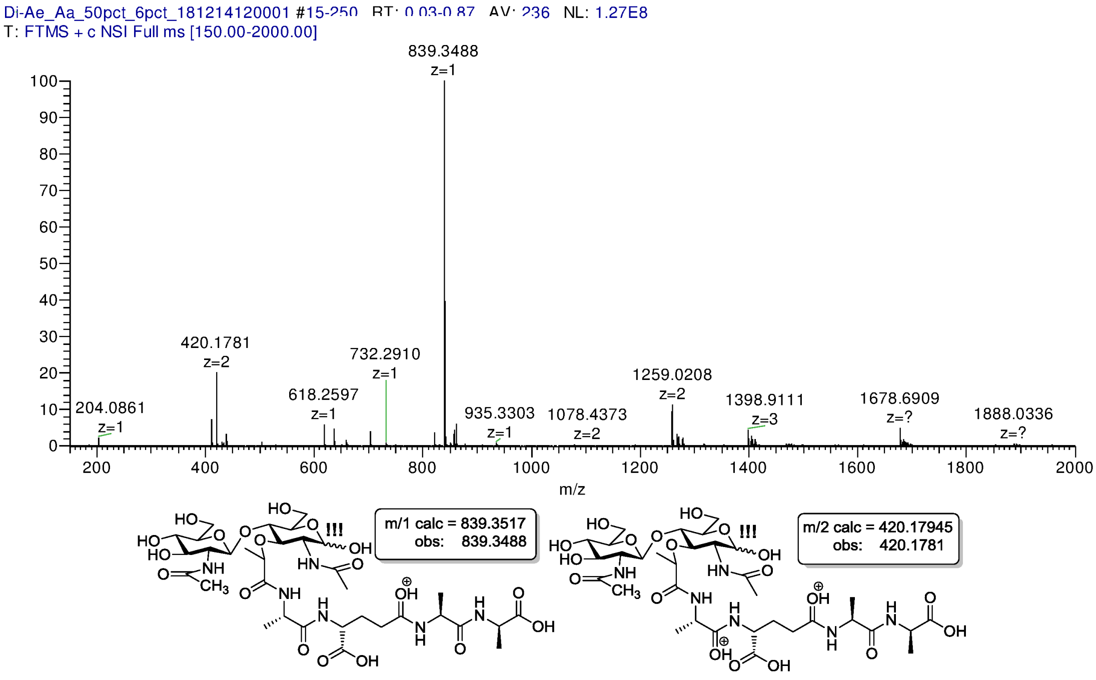

Figure 9: N-Acetylglucosaminyl-β-(1-4)-N-acetylmuramoyl-L-Alanyl-γ-D-glutamyl-L-alanyl-D-alanine (**1**). HRMS spectrum.
